# The rate of photosynthetic induction across different light intensities can be approximated using the light response curve of photosynthesis

**DOI:** 10.1007/s11120-026-01216-2

**Published:** 2026-04-24

**Authors:** Elias Kaiser, Ningyi Zhang, Sarah Berman, Mehrdad Behzadian, Silvere Vialet-Chabrand, Leo F. M. Marcelis

**Affiliations:** 1 Horticulture and Product Physiology, Plant Sciences Group, Wageningen University, Wageningen, The Netherlands; 2 Research Institute of Agriculture and Life Sciences, Seoul National University, Seoul, Republic of Korea; 3 College of Horticulture, Nanjing Agricultural University, Nanjing, China

**Keywords:** Photosynthetic induction, PPFD, Light response curve, Carbon dioxide, Air humidity

## Abstract

While natural light in plant canopies often fluctuates and shows a large range of possible intensities, the rate of photosynthetic induction is often measured at only two light intensities. This prompts the question as to how the relationship between different light intensities and the rate of photosynthetic induction can be characterized. Using hundreds of measurements of photosynthetic induction at various light intensities in leaves of tomato (*Solanum lycopersicum*), we found linear relationships between the time to reach 50% of full photosynthetic induction (t_50_) and the difference between steady-state photosynthesis rates at low and at high light intensities (Δ*A*). This relationship was strongest at low leaf-to-air vapour pressure deficits (0.5-1.0 kilopascal), and when dark-adapted leaves were excluded. This relationship was documented at different CO_2_ mole fractions, namely 400 (R^2^ = 0.86), 600 (R^2^ = 0.66) and 800 µmol mol^− 1^ (R^2^ = 0.92). A survey of the literature suggested that this relationship also exists in various other tree and herbaceous species. The t_50_- Δ*A* relationship is particularly useful as proxy for the rate of photosynthetic induction between any two light intensities, through the use of conventional steady-state photosynthesis light response curves. A guide for best practice for characterizing the t_50_- Δ*A* relationship in the leaves of interest is provided.

**Supplementary Information:**

The online version contains supplementary material available at 10.1007/s11120-026-01216-2.

In nature, the light that strikes the leaves inside a canopy is often unstable and can show thousands of transitions from one light intensity to another per day (Durand and Robson 2023). Transitions from a low to a high light intensity are termed sunflecks, and the reverse shadeflecks. Photosynthesis in shade-adapted leaves reacts to sunflecks with a delay, presenting a potential inefficiency that crop breeding and synthetic biology can act upon to improve productivity and resource use efficiency (Long et al. 2022). Kinetics of photosynthesis during sunflecks are often quantified by measuring photosynthetic induction, i.e. the rate of increase in leaf net photosynthesis rate (*A*) after a switch from a low (Q_L_) to a high (Q_H_) light intensity (Fig. 1A), and then calculating the time required to reach 50 and 90% of photosynthetic induction (t_50_, t_90_; Fig. 1B). The rate of photosynthetic induction is chiefly controlled by the activation state of key enzymes in the Calvin-Benson-Bassham cycle, and by the two conductances to CO_2_ diffusion within the leaf, stomatal conductance (*g*_*s*_) and mesophyll conductance (Acevedo-Siaca and McAusland 2025).

**Fig. 1:**
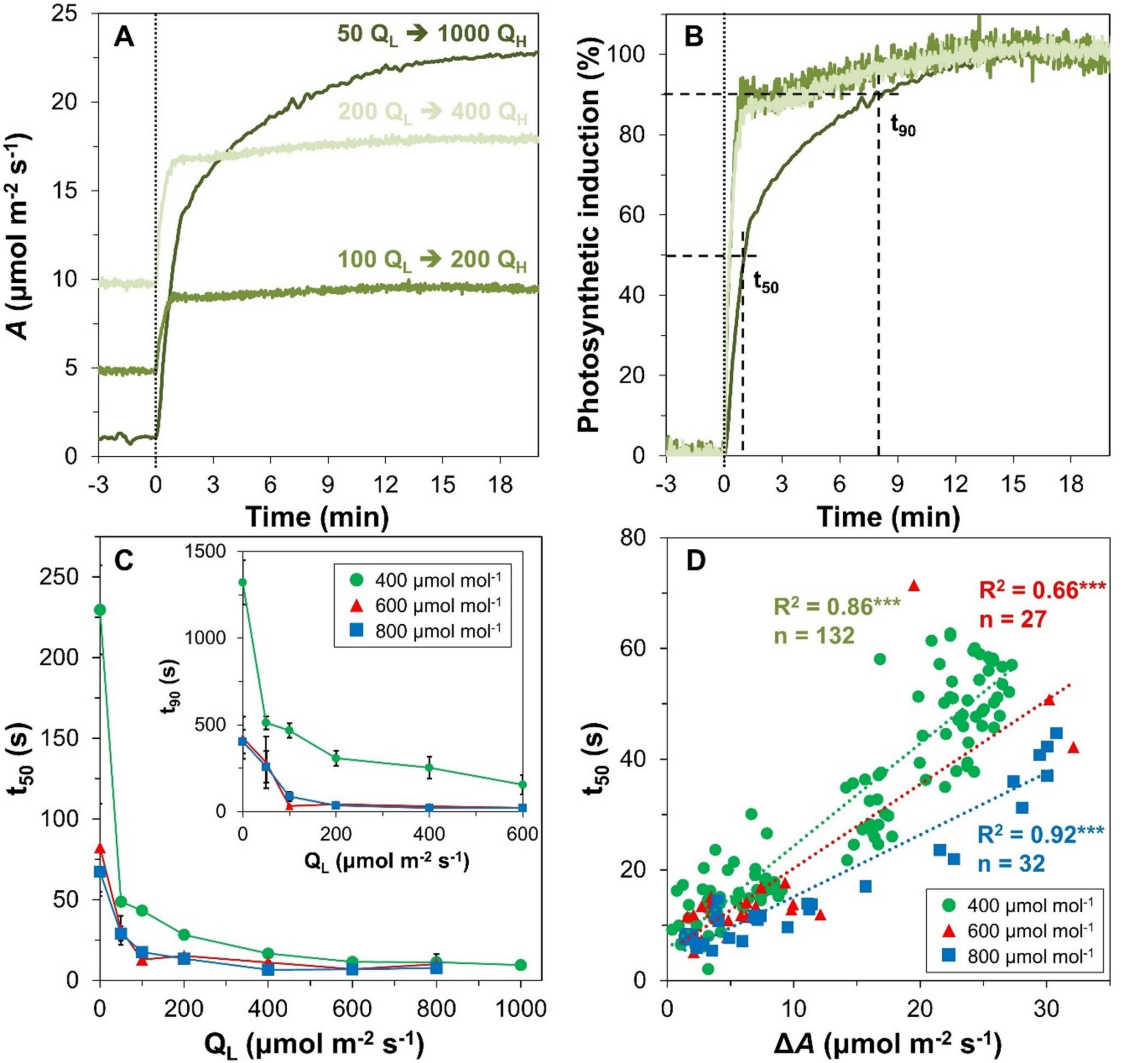
The rate of photosynthetic induction across light intensities scales with net photosynthesis rate in tomato leaves. **A**) Examples of time courses of net photosynthesis rate (*A*) after shifts from various low (Q_L_) to various high light intensities (Q_H_); **B**) time courses of photosynthetic induction (data from panel A normalized by initial and final *A*). Times to reach 50 (t_50_) and 90% (t_90_) of final steady-state photosynthesis are indicated. **C**) Relationships between t_50_ and Q_L_ across a large range of Q and three [CO_2_]; inset: relationships between t_90_ and Q_L_; averages ± standard errors of means (SEM) are shown (*n* = 2–33), unless SEM are smaller than symbols of means. **D**) Relationships between t_50_ and the difference in steady-state *A* under high and low light intensity, (Δ*A*), across a large range of Q and three [CO_2_]. Dotted lines in panels A and B mark the moment of light intensity shift. In panel D, Pearson correlation coefficients (R^2^) and number of datapoints (n) are shown, stars indicate *p* < 0.001 in each case; datasets from initially dark-adapted leaves (Q_L_ = 0 µmol m^− 2^ s^− 1^) are excluded

The photosynthetic induction state (i.e., the degree of activation of photosynthesis) depends on light intensity. Both Q_L_ and Q_H_ show tremendous variability from sunfleck to sunfleck under natural conditions. However during experiments on photosynthetic induction, the role of different light intensities is often ignored: in a given experimental setup, photosynthetic induction is typically assessed using one value for Q_L_ and Q_H_ each. Typically, leaves are initially adapted to a Q_L_ that is very low (often: 0-100 µmol m^− 2^ s^− 1^), and are then exposed in a stepwise change to a Q_H_ that is near-saturating (often approx. 1000 µmol m^− 2^ s^− 1^). Under these conditions, the rate of photosynthetic induction is slow. However, different combinations of Q_L_ and Q_H_ strongly affect the rate at which *A* increases (Fig. 1A; Carmo-Silva and Salvucci 2013) and at higher Q_L_ (such as often occurs in nature), the rise in *A* can be very rapid (Fig. 1A, B). This means that depending on the value of Q_L_ (and possibly Q_H_), the time constant for the rise of *A* changes, but this fact is typically ignored. The time constant of photosynthetic induction is the product of multiple processes (such as Rubisco activation state and stomatal opening), each having their own time constants and light intensity dependences. The widespread use of such static Q_L_-Q_H_ combinations across studies causes at least three problems in the scientific literature: (1) it leads to the false conclusion that the rate at which *A* increases after a step increase in Q is generally slow, (2) it suggests unrealistically large inefficiencies in dynamic photosynthesis under natural conditions, and (3) it precludes realistic yet simple models of leaf photosynthesis dynamics under natural fluctuations in light intensities, such as occur in crop canopies.

If the time constant of photosynthetic induction is really a function of Q_L_ and Q_H_, simple models for the rate of photosynthetic induction may become very tedious to parameterize, as this would require the measurement of photosynthetic induction under a large number of combinations in Q_L_ and Q_H_, which is highly impractical. However, given that *A* increases nonlinearly with Q in the well-known *A*/Q response curve (Fig. 2A) while t_50_ and t_90_ have been shown to decline exponentially with increases in Q_L_ (Kaiser et al. 2017a, b linear relationship between steady-state *A* and these time constants may exist. Such a relationship would simplify the estimation of time constants of photosynthetic induction across different light intensities.

To probe the relationship between the rate of photosynthetic induction and light intensity, tomato (*Solanum lycopersicum* cv. Merlice) leaves were exposed to hundreds of step increases in Q over a large range of Q_L_ (0-1000 µmol m^− 2^ s^− 1^) and Q_H_ (50-1200 µmol m^− 2^ s^− 1^), resulting in 407 time courses of photosynthetic induction. Photosynthetic induction was measured at different levels of CO_2_ mole fraction ([CO_2_]) and leaf-to-air vapour pressure deficit (VPD), and datasets were grouped by VPD as: 0.5-1.0 kPa, 1.01–1.5 kPa, and 1.51–2.4 kPa. Datasets were further subdivided into [CO_2_] of 400, 600 and 800 µmol mol^− 1^ (Table S1; see supplementary methods for more details). Unless specifically mentioned, the results below describe findings for the 0.5-1.0 kPa VPD group.

**Fig. 2:**
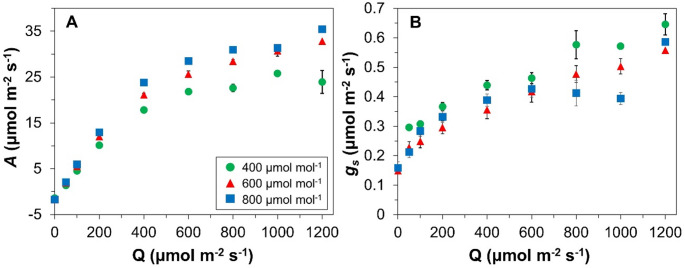
Light response curves of (**A**) net photosynthesis rate (*A*) and of (**B**) stomatal conductance (*g*_*s*_) at three [CO_2_]. Data were aggregated from steady states of *A* and g_s_ measured under both Q_L_ and Q_H_ as part of various photosynthesis induction measurements. Data show averages (*n* = 1-127), and show standard errors of means when *n* > 1

Both t_50_ and t_90_ showed strong negative relationships with Q_L_, and dark-adapted leaves (Q_L_: 0 µmol m^− 2^ s^− 1^) showed much slower photosynthetic induction than shade-adapted leaves (Fig. 1C). Crucially, t_50_ showed highly significant (*p* < 0.001) linear relationships with Δ*A*, with R^2^ ranging from 0.66 to 0.92 across different [CO_2_] (Fig. 1D). The slope of the t_50_-Δ*A* relationship became shallower with increasing [CO_2_], suggesting that at higher [CO_2_], t_50_ was reached more quickly for a given Δ*A*. Data retrieved from prior studies in rice (Fukuyama et al. 1998), *Arabidopsis thaliana* (Kaiser et al. 2016), tomato cv. Cappricia (Kaiser et al. 2017b) and Japanese beech (*Fagus crenata*) (Han et al. 1999; Naramoto et al. 2001) broadly confirmed positive t_50_-Δ*A* relationships (Fig. 3A), with much steeper t_50_-Δ*A* relationships being found for tree (Fig. 3B) than for herbaceous species (Fig. 3A). Further, datasets derived from (Kaiser et al. 2017b) confirmed the observed flattening of the t_50_-Δ*A* relationship with increasing CO_2_ partial pressure. When plotting slopes from both datasets presented in Figs. 1D and 3A against CO_2_ mole fraction, a common pattern emerged (Fig. S1). Altogether, these data imply that while a positive t_50_-Δ*A* relationship generally exists in various C_3_ species, their steepness depends on (at least) species and CO_2_ mole fraction during photosynthesis measurements.

**Fig. 3:**
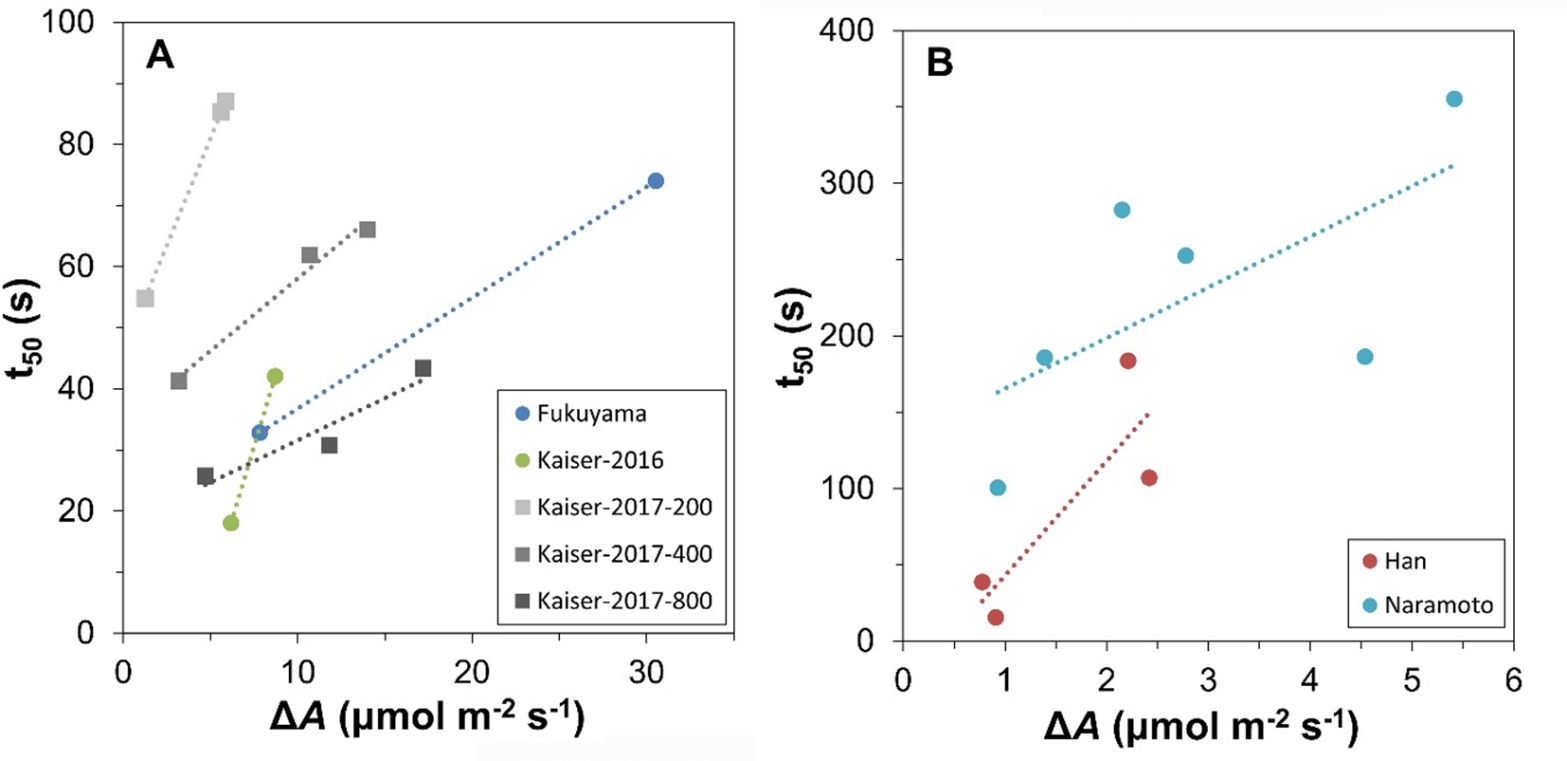
Relationships between the time to reach 50% of full photosynthetic induction (t_50_) and the difference in *A* under high and low light intensity (Δ*A*), in a range of studies. **A**) herbaceous species: rice (Fukuyama et al. 1998), *Arabidopsis thaliana* Col-0 (Kaiser et al. 2016), tomato cv. Cappricia (Kaiser et al. 2017a, b). **B**) tree species: Japanese beech (*Fagus crenata*; (Han et al. 1999; Naramoto et al. 2001). In each study, at least two different low (Q_L_) and/or high (Q_H_) light intensities were used; these ranged from 25 to 500 µmol m^− 2^ s^− 1^ for Q_L_, and from 240 to 1800 µmol m^− 2^ s^− 1^ for Q_H_. For most datasets, CO_2_ mole fraction [CO_2_] was in the range 350–400 µmol mol^− 1^, except for “Kaiser-2017-200” (200 µmol mol^− 1^) and “Kaiser-200-800” (800 µmol mol^− 1^). Note that panels A and B differ in the range of x- and y-axis values

By comparison with t_50_, regressions between Δ*A* and t_90_ were much more scattered (R^2^ = 0.26–0.54), although they were nevertheless highly significant (Fig. S2). It should be stressed that the linear relationships between Δ*A* and t_50_ (Fig. 1D) and Δ*A* and t_90_ (Fig. S1) existed only when excluding measurements on dark-adapted leaves (i.e. Q_L_ ≥ 50 µmol m^− 2^ s^− 1^); when these were included, data looked much more noisy, with much larger values for t_50_ and t_90_ (Fig. S3). This again confirms (Kaiser et al. 2017b) that dark-adapted leaves show much slower photosynthetic induction than low light-adapted leaves. Slower photosynthetic induction in dark-adapted leaves is likely caused by a combination of lower initial Rubisco activation state, slower Rubisco activation, lack of intermediates in the Calvin cycle, and lower *g*_*s*_.

Stomatal conductance (*g*_*s*_) typically increases with rising Q (Fig. 2B), and thus also during photosynthetic induction. Because low *g*_*s*_ limits the rate of CO_2_ diffusion into the leaf, the rate of photosynthetic induction often shows a biphasic relationship with *g*_*s*_ before the switch in Q (*g*_*s*L_) (Kaiser et al. 2016). Despite this often documented relationship, in our dataset we observed weaker relationships between *g*_*s*L_ and t_50_ or t_90_ (Fig. S4) compared to those between Δ*A* and t_50_ or t_90_. Finally, we found that at high VPD (1.0-2.4 kPa), the relationships between Δ*A* and t_50_ or t_90_ were much more scattered than at lower VPD (Fig. S5), partially due to stomatal oscillations at high VPD that affected the rate at which a stable *A* was reached (Kaiser et al. 2017a).

In conclusion, our data reveal clear linear relationships between the rate of photosynthetic induction (when this was represented by t_50_) and steady-state photosynthesis rates, across a range of light intensities. These relationships were found to be solid under low VPD (0.5-1.0 kPa), and when excluding dark-adapted leaves. The positive relationship between t_50_ and Δ*A*, as well as the positive effect of [CO_2_] on its slope, could be partially explained by the fact that some of the energy required to activate key photosynthetic enzymes is generated through photosynthesis, so higher *A* could potentially lead to faster responses when light intensity is increased. However, a more thorough analysis of biochemical activation states, and limitations by e.g. stomatal and mesophyll conductance, should be conducted to test this hypothesis. These observed relationships are elegant, as they reduce a three-dimensional relationship (between Q_L_, Q_H_, and t_50_) to one that is two-dimensional (between Δ*A* and t_50_). Furthermore, these relationships are comparably practical, as a *A*/Q curve and a few photosynthesis induction measurements are in principle sufficient to estimate t_50_ for induction at different Q. In Supplementary Methods 2, we formulate a guide to best practices for characterizing the t_50_-Δ*A* relationship that readers can use. This relationship could help in the construction of simple models of dynamic photosynthesis, which would not have to explicitly model the kinetics of underlying limitations, such as those of Rubisco activation state and g_s_ (e.g. Morales et al. 2018). It should be kept in mind that in real crop canopies, leaves are often induced from irradiance levels below 50 µmol m⁻² s⁻¹, especially in the middle and lower canopy. Likewise, VPD values above 1.0 kPa, and often above 1.5 kPa, are common under field conditions. Furthermore, in this study we only present data from a few C_3_ species, and it should be explored whether other plant functional types as well as photosynthesis pathways (C_4_, CAM) show the same relationship. Regardless, the results presented here provide an important and useful empirical approximation between dynamic and steady-state photosynthesis rates that works well under narrowly defined experimental parameters.

## Electronic supplementary material

Below is the link to the electronic supplementary material.

Supplementary Material 1

## Data Availability

Data is available upon reasonable request.
