# Supplementary material for: The rate of photosynthetic induction across different light intensities can be approximated using the light response curve of photosynthesis
Source: Photosynth Res. 2026 Apr 24;164(3):28. doi: 10.1007/s11120-026-01216-2 (PMC13109170; doi:10.1007/s11120-026-01216-2)
Supplement: Supplementary file 1 — Supplementary Material 1 [file 11120_2026_1216_MOESM1_ESM.docx]

**Supplementary material to: “The rate of photosynthetic induction across different light intensities can be approximated using the light response curve of photosynthesis”**

Elias Kaiser, Ningyi Zhang, Sarah Berman, Mehrdad Behzadian, Silvere Vialet-Chabrand, Leo F.M. Marcelis

**Supplementary methods 1**

Plant materials and growth conditions

Tomato seeds (cv. ‘Merlice’; Bayer, the Netherlands) were sown in rockwool plugs (Grodan, Roermond, NL), and were transplanted to 10 x 10 x 7 cm rockwool cubes (Grodan) one week after sowing. Plants were grown in a climate chamber with dimmable LED modules (DRWFR_RSE 400 V 1.1DMP, Signify, NL). The light spectrum was 5.1% blue, 8.9% green, 77.2% red, and 8.8% far-red. Dynamic changes in the extent of dimming of the LED modules created a fluctuating light regime that was intended to mimic natural fluctuations in light intensity. The exact pattern of the growth light regime was randomized daily; however, the following parameters were kept identical between days: the photoperiod was 16 h, the average, minimum and maximum light intensity were 250, 120 and 1200 μmol m^− 2^ s^− 1^, respectively, and the overall shape of the diurnal light pattern was sinusoidal (for more details on the light pattern, see (Shrestha *et al.* 2025)). Day and night temperatures were set to 23 and 20°C, respectively, relative air humidity (RH) was set to 70%, and [CO_2_] was ambient (ca. 430 μmol mol^− 1^). Plants were irrigated with a standard nutrient solution twice daily using an automatic ebb- and flood system (for details see Zhang et al., 2024).

Data acquisition

Hundreds of measurements of leaf photosynthetic gas exchange were conducted using the Li-6800 photosynthesis system (Li-Cor Biosystems, Lincoln, Nebraska, USA); in about 10% of cases, the Li-6400 photosynthesis system was used (Li-Cor Biosystems). In all cases, the fluorometer cuvette was used as light source. Measurements were conducted in the first half of the photoperiod, usually between 9:00 and 17:00. Fully expanded leaves on plants that were 4–5 weeks old were used; measurements were conducted on the two leaflets that were located on either side of the terminal leaflet. All measurements were conducted at 90% red and 10% blue light and a flow rate of 500 μmol s^− 1^, as well as minimal instrument averaging time. Leaf temperature (T_leaf_) ranged from 22.5–26.3°C, with an average ± SEM of 24.6 ± 0.1°C. Leaf-to-air vapour pressure deficit (VPD) ranged from 0.5 to 2.4 kPa, with an average ± SEM of 1.03 ± 0.01.

Measurements of photosynthetic induction were conducted at various low (Q_L_) and high light intensities (Q_H_), and combinations thereof. Q_L_ included intensities of 0, 50, 100, 200, 400, 600, 800, and 1000 μmol m^− 2^ s^− 1^, while Q_H_ included intensities of 50, 100, 200, 400, 600, 800, 1000 and 1200 μmol m^− 2^ s^− 1^. Each measurement resulted in one set of raw data (hereafter: raw dataset) comprising a unique time course of Q, *A*, g_s_, VPD, T_leaf_, [CO_2_], and flow rate. In each raw dataset, Q_L_ was by definition lower than Q_H_. Furthermore, [CO_2_] in the reference chamber was set to either 400, 600 or 800 μmol mol^− 1^. Leaves were initially adapted to Q_L_ until *A* and g_s_ were stable (at least 30 minutes), and were then exposed to Q_H_ in a stepwise change, until *A* was stable; stability was verified by visual inspection of the data. Data were logged every 1–2 seconds. Infra-red gas analysers were matched prior to each measurement.

Previously published data of photosynthetic induction at various light intensities (i.e., time courses of *A*) were digitized using WebPlotDigitizer (version 3.8). Criteria for inclusion were that i) there were at least two Q_L_ and/or Q_H_ levels per species studied, and ii) Q_L_ was > 0 μmol m^− 2^ s^− 1^. This yielded 23 datasets from five studies (Fukuyama, Uchida, Azuma & Yasuda 1998; Han Q., Yamaguchi E., Odaka N. & Kakubari Y. 1999; Naramoto M., Han Q. & Kakubari Y. 2001; Kaiser *et al.* 2016, 2017). Data were then analyzed as described below.

Data analysis

Each raw dataset was at first inspected in Excel, and data rows showing excessive noise (such as sudden ‘jumps’ in *A* or g_s_) were deleted. Raw datasets with very low signal/noise ratios, such as can occur at very small light intensity steps in the near-saturating range of the *A*/Q curve (e.g. step change in Q from 800 to 1000 μmol m^− 2^ s^− 1^), were discarded. Also, raw datasets where *A* showed a reduction after reaching a transient maximum value under Q_H_ (this was often caused by reductions in g_s_ under Q_H_) were discarded. In total, 407 out of 505 raw datasets (81%), plus 23 published datasets, were used in the subsequent analysis.

Raw datasets were imported into Rstudio (Posit PBC, Boston, Massachusetts, USA). Averages of Q, *A*, g_s_, VPD, T_leaf_, [CO_2_], and flow rate under Q_L_ and Q_H_ were calculated. A local polynomial regression using the ‘loess’ function was then fitted to *A*, to interpolate between measured data and to smoothen fluctuations in measured data. Span, a parameter controlling the degree of smoothing, was set to 5% of the input data, and the root mean squared error (RMSE) between modelled and measured data was calculated. For a detailed discussion of the ‘loess’ function and the use of span, see (Simpson & Haggard 2018). RMSE was 0.008 μmol m^− 2^ s^− 1^ on average across all raw datasets, ranging from 0.0017 to 0.017 μmol m^− 2^ s^− 1^, and was thus very small relative to absolute changes in *A*. A new time series of *A* as predicted by loess was then constructed per raw dataset at a resolution of 0.05 s (i.e., 20 values per second), and t_50_ and t_90_ were estimated using this time series. Per raw dataset, RMSE, t_50_, t_90_, Q_L_, Q_H_, *A*_L_, *A*_H_, Δ*A*, *g_s_*_L_, *g_s_*_H_, VPD, [CO_2_], flow rate and T_leaf_ were recorded, and compiled into a new spreadsheet of processed data (hereafter: processed datasets). Processed datasets were divided into three groups as a function of VPD: 0.5-1.0 kPa, 1.01–1.5 kPa, and 1.51–2.4 kPa, then subdivided by [CO_2_], and further divided by Q_L_ (0 vs. ≥50 μmol m^− 2^ s^− 1^; see Table S1 for number of processed datasets per category).

Standardized major axis analysis was used in R (function: ‘sma’) to obtain Pearson’s R^2^ and the *p*-value of linear regressions between Δ*A* and t_50_ as well as t_90_ at 400, 600 and 800 μmol mol^− 1^ [CO_2_], respectively.

**Supplementary methods 2**

A guide to best practices for determining the t_50_-Δ*A* relationship

As our results demonstrate, the t_50_-Δ*A* relationship depends on (at least) species, CO_2_ mole fraction, VPD and initial light intensity (Q_L_) during measurements (Fig. 1D, 3, S3). Therefore, this relationship should be determined for each given set of experimental plants, and its limitations should be kept in mind when interpreting the data or when including a representation of the relationship in dynamic photosynthesis models. The following steps should be taken to produce reliable results:

1. Determine conventional light response curves of photosynthesis (*A*/Q curve), on at least three independent biological replicates. After reaching steady-state *A* at the highest light intensity, determine *A* in quick steps (ca. 2 min per step) from the highest to the lowest light intensity (e.g. 2000, 1500, 1000, 800, 600, 400, 300, 200, 150, 100, 70, 30, 0 μmol m^-2^ s^-1^). Each curve will take ~ 35–90 min, depending on initial light adaptation state of the plant material to be used.
2. Using results from step 1, determine the light intensity at which ca. 90% of full light saturation is reached. *As an example, let’s assume that this light intensity is 1000 μmol m^-2^ s^-1^.*
3. Using results from step 1, determine the light compensation point, i.e. the light intensity at which *A* = 0 μmol m^-2^ s^-1^. *As an example, let’s assume that this light intensity is 50 μmol m^-2^ s^-1^.*
4. The most effective range for photosynthesis induction measurements is between the light compensation point and the light intensity at which ca. 90% of full light saturation is reached *(i.e., 50-1000 μmol m^-2^ s^-1^ in our example)*.
5. Within this range, pick at least five pairs of light intensities (Q_L_, Q_H_) for induction measurements. These should be chosen such that they produce a measurable *Δ*A, and that the five values of *Δ*A distribute well along the x-axis of the t_50_-*Δ*A relationship to be determined from these data (see Fig. 1D). The *A*/Q curve from step 1 can help in determining suitable pairs of Q_L_ and Q_H_.
6. Measure photosynthetic induction on (at least) three independent statistical replicates per Q_L_-Q_H_ pair (more replicates and/or Q_L_-Q_H_ pairs can be added to increase confidence, at additional time investment). For best results, adapt the leaf at Q_L_ until stomatal conductance (g_s_) is stable (this may take ~ 30–120 min), then switch to Q_H_ and log (as quickly as the gas exchange equipment allows) until *A* is stable. Each photosynthetic induction measurement will take ~ 60–180 min, depending on how quickly steady-state *A* and g_s_ are reached
7. Calculate t_50_ and *Δ*A from the obtained photosynthesis time courses. Plot all together (such as in Fig. 1D) to obtain slope and intercept of the t_50_-*Δ*A relationship.
8. Using the *A*/Q curve, calculate t_50_ for any desired combination of Q_L_ and Q_H_ (via *Δ*A)

**Table S1.** Number of processed datasets per category. Datasets were categorized by leaf-to-air vapour pressure deficit (VPD), [CO_2_], and low (initial) light intensity (Q_L_).

| Factor |  |  |  |
| --- | --- | --- | --- |
| VPD (kPa) | [CO_2_] (μ μmol mol^− 1^) | Q_L_ (μmol m^− 2^ s^− 1^) | No. processed datasets |
| 0.5-1.0 | 400 | ≥ 50 | 132 |
| 0.5-1.0 | 600 | ≥ 50 | 27 |
| 0.5-1.0 | 800 | ≥ 50 | 32 |
| 1.01–1.5 | 400 | ≥ 50 | 54 |
| 1.01–1.5 | 600 | ≥ 50 | 45 |
| 1.01–1.5 | 800 | ≥ 50 | 42 |
| 1.51–2.4 | 400 | ≥ 50 | 3 |
| 1.51–2.4 | 600 | ≥ 50 | 4 |
| 1.51–2.4 | 800 | ≥ 50 | 9 |
| 0.5-1.0 | 400 | 0 | 18 |
| 0.5-1.0 | 600 | 0 | 7 |
| 0.5-1.0 | 800 | 0 | 7 |
| 1.01–1.5 | 400 | 0 | 7 |
| 1.01–1.5 | 600 | 0 | 4 |
| 1.01–1.5 | 800 | 0 | 9 |
| 1.51–2.4 | 400 | 0 | 2 |
| 1.51–2.4 | 600 | 0 | 3 |
| 1.51–2.4 | 800 | 0 | 2 |


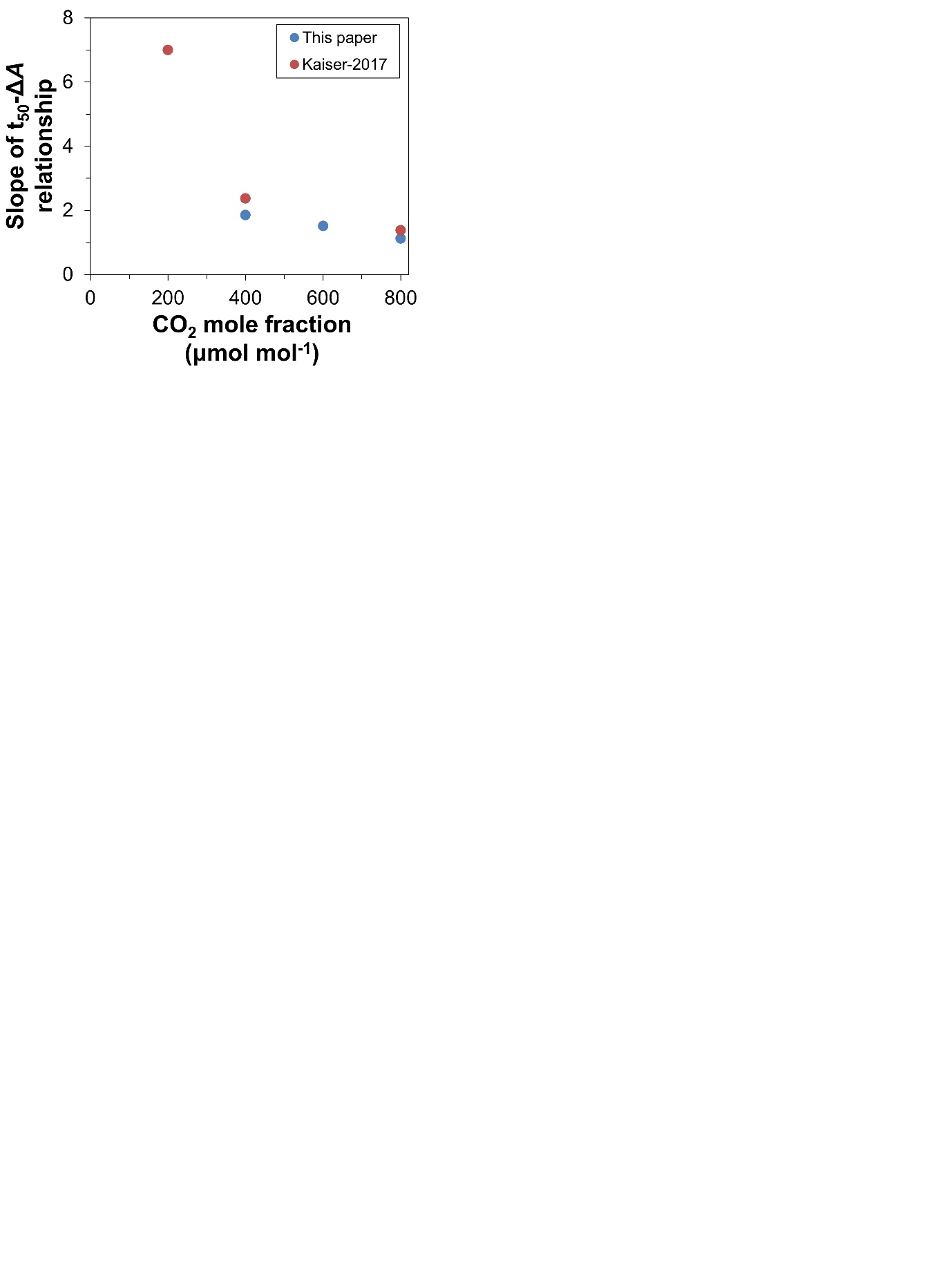


**Fig. S1.** Relationship between the slope of the t_50_-Δ*A* relationship and CO_2_ mole fraction during photosynthetic induction. Blue symbols are slope values originating from this study (Fig. 1D), red symbols originate from the relationships shown in Fig. 3A, with original data from (Kaiser *et al.* 2017).


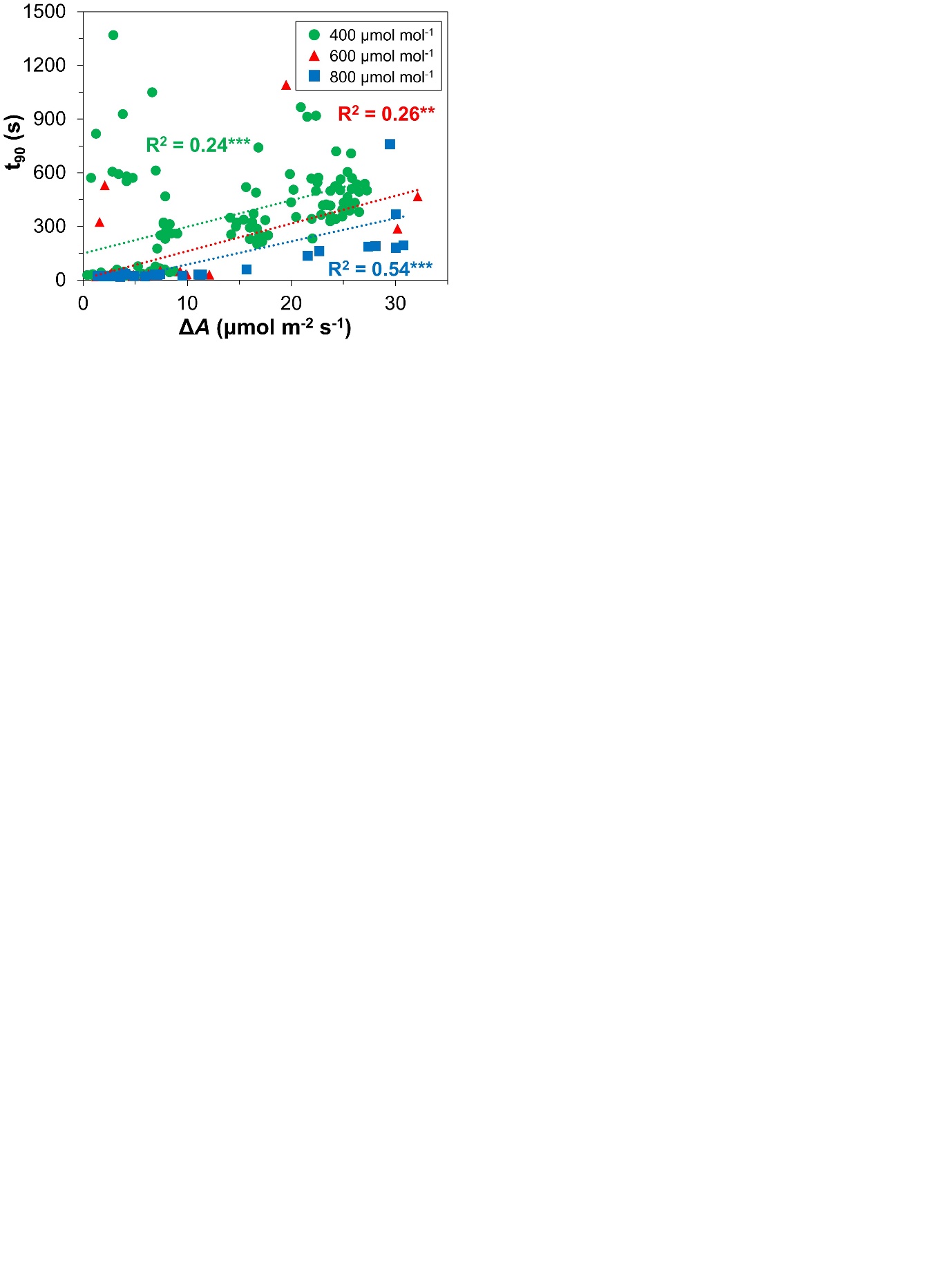


**Fig. S2.** Relationships between the time to reach 90% of full photosynthetic induction (t_90_) and the difference in *A* under high and low light intensity (Δ*A*), across a large range of Q and three [CO_2_]. Note that data with initially dark-adapted leaves (Q_L_ = 0 μmol m^− 2^ s^− 1^) are not shown. Pearson correlation coefficients (R^2^) are shown; stars indicate ** = *p* < 0.01 and *** = *p* < 0.001, respectively.


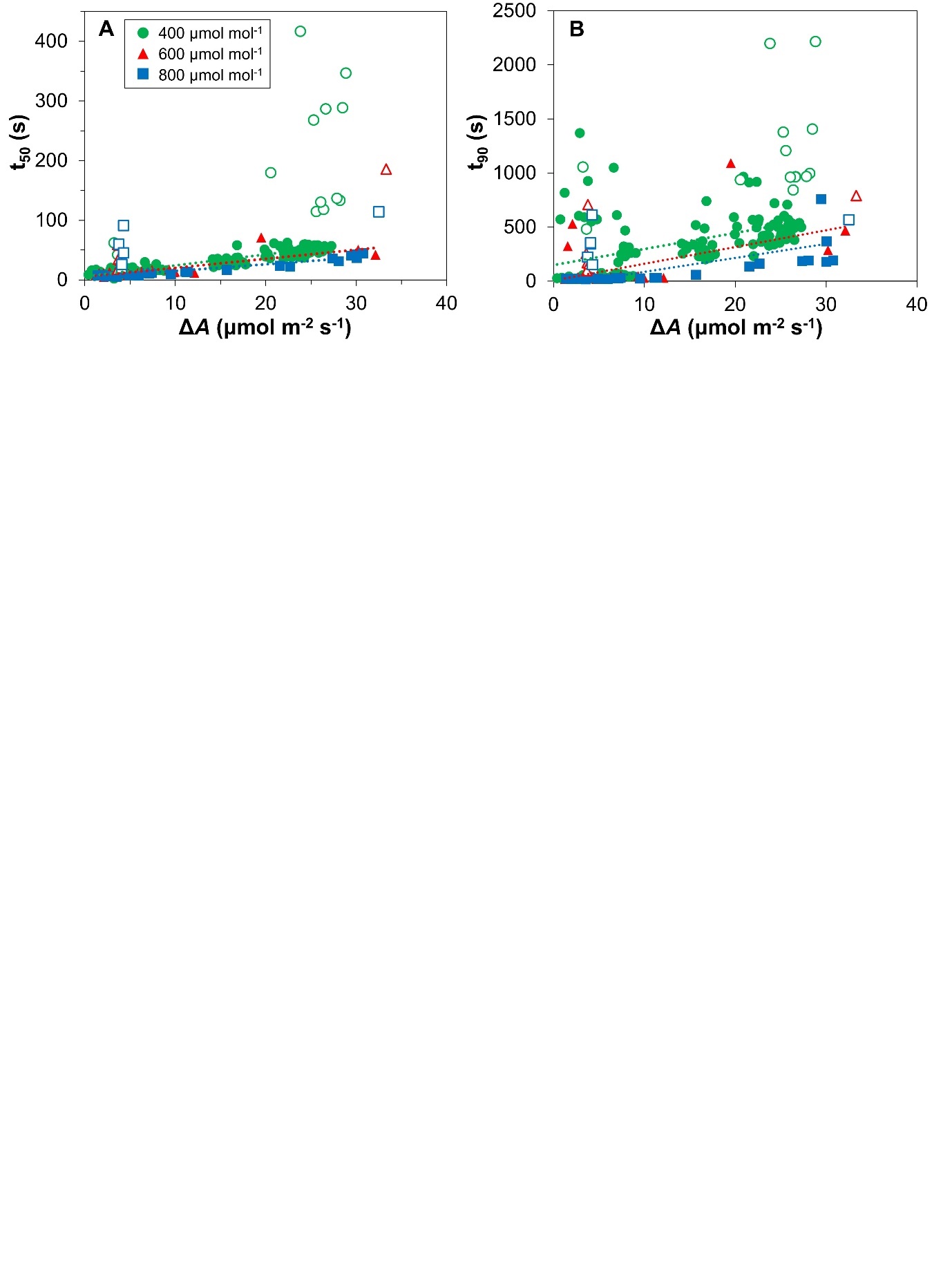


**Fig. S3.** Relationships between the difference in *A* under high and low light intensity (Δ*A*) and the time to reach 50 (t_50_, A) or 90% (t_90_, B) of full photosynthetic induction, across a large range of light intensities, and at three [CO_2_]. Empty symbols indicate initially dark-adapted leaves (Q_L_ = 0 μmol m^− 2^ s^− 1^) before the shift in light intensity, filled symbols indicate Q_L_ ≥50 μmol m^− 2^ s^− 1^. Regression lines only include filled symbols.

**
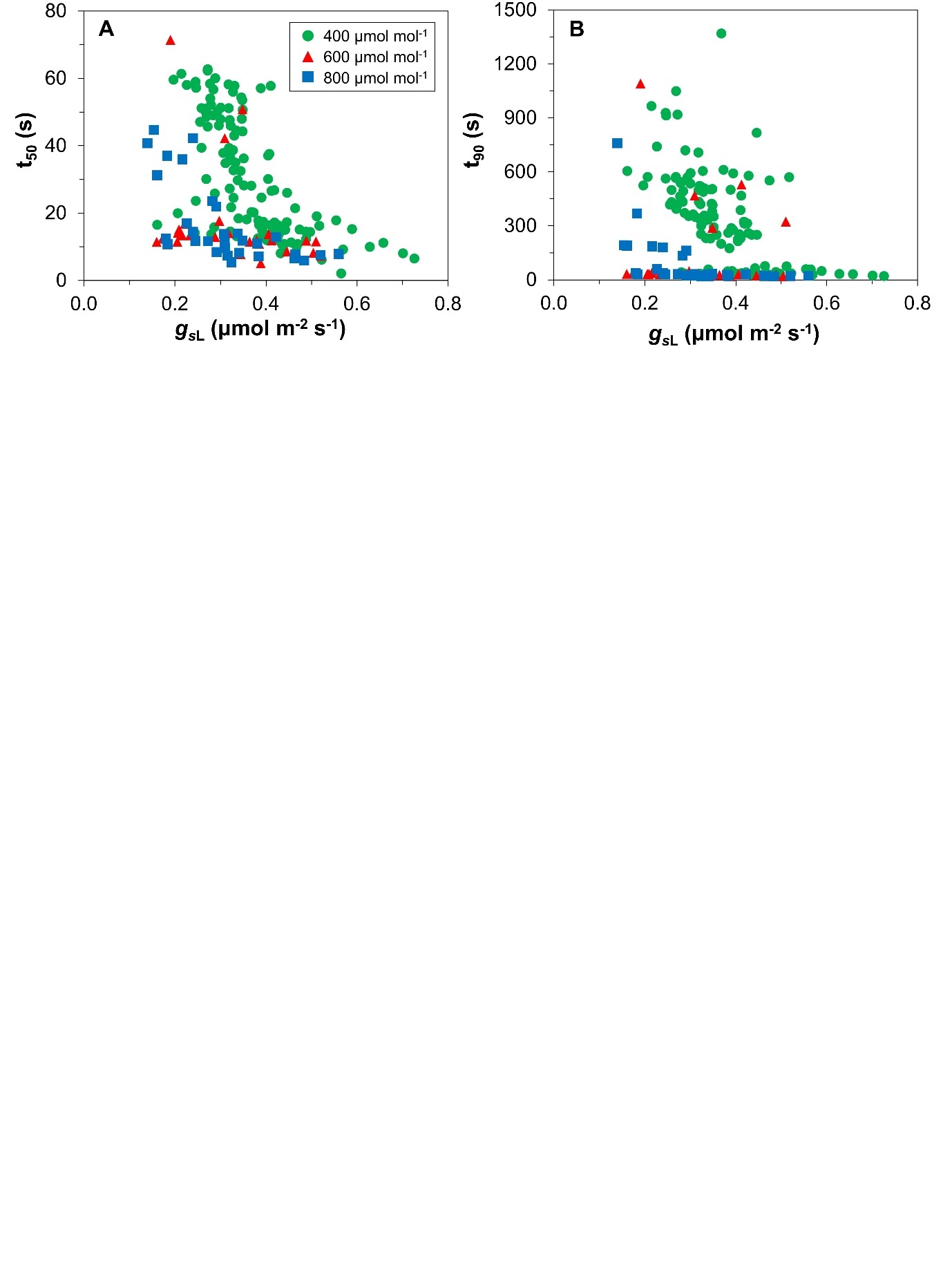
**

**Fig. S4.** Relationships between stomatal conductance at low light intensity (*g_s_*_LL_) and the time to reach 50 (t_50_, A) or 90% (t_90_, B) of full photosynthetic induction across a large range of light intensities, and at three [CO_2_].


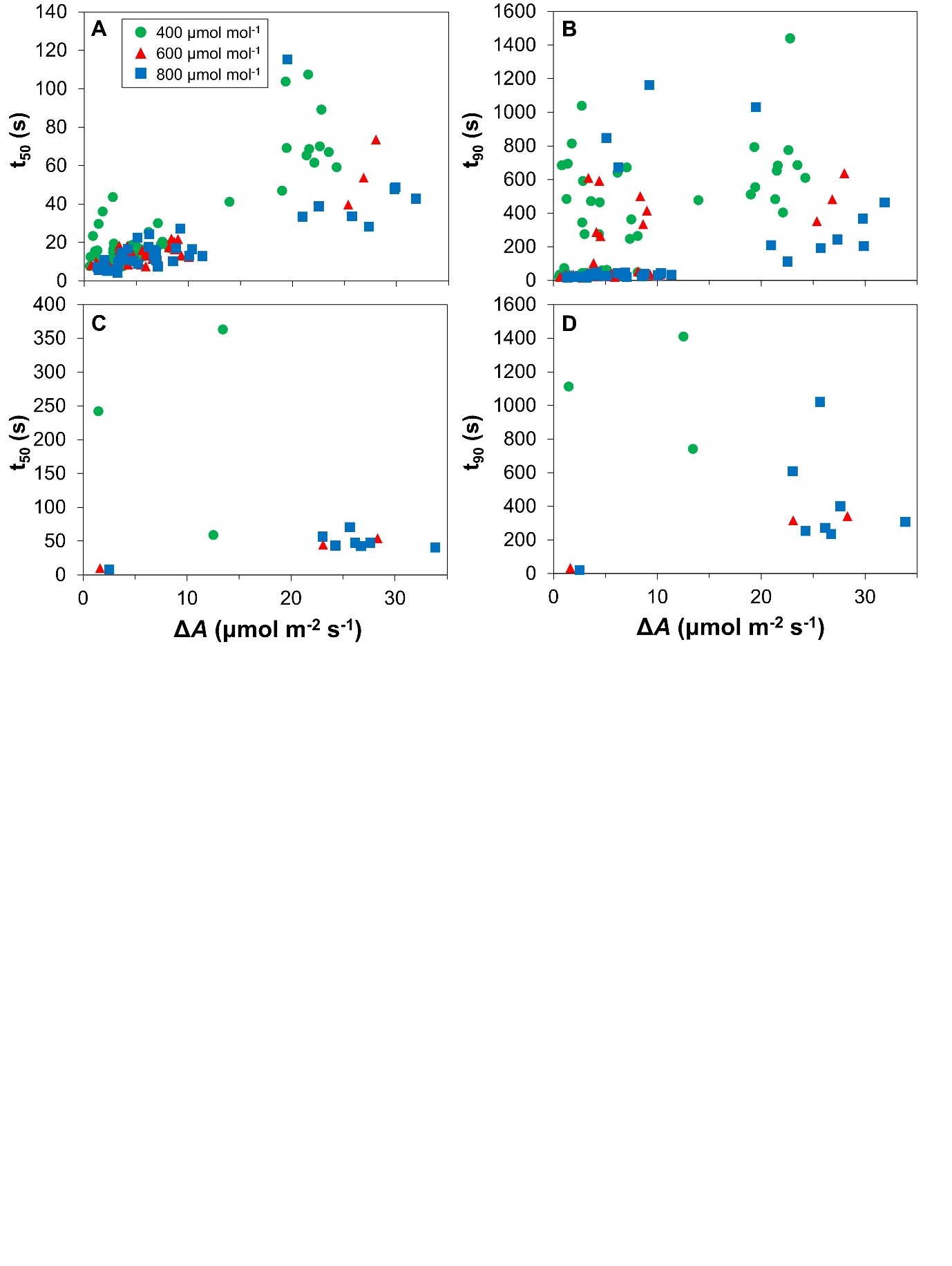


**Fig. S5.** Relationships between the difference in *A* under high and low light intensity (Δ*A*) and the time to reach 50 (t_50_, A) or 90% (t_90_, B) of full photosynthetic induction at high leaf-to-air vapour pressure deficits (VPD), and at three [CO_2_]. Panels A and B show data at 1.01–1.5 kPa VPD, panels C and D show data at 1.51–2.4 kPa.
